# On the Treatment of Burns and Scalds

**Published:** 1807-05

**Authors:** 


### *To the Editors of the Medical and Physical Journal.*

GENTLEMEN,

THE controversy, which has occupied so many pages in your Journal, respecting the Treatment of Burns and Scalds, appears to have been persevered in by the advocates for the stimulant practice, from a total mis-apprehension of the import of Dr. Kentish's experience in that method of cure. Convinced that the judicious application of COLD is the only means by which we can *considerably and instantaneously* alleviate the extreme severity of these distressing accidents, I cannot forbear exposing the fallacies, which have long been persisted in, for the purpose of preventing the general employment of this important remedy. Sir James Earle, Sir Walter Farquahar, and of late, Doctors Kinglake, Hall, and others, have all insisted upon the supreme excellence of cold, when made use of at proper periods, in these injuries; and the numerous cases, which these Gentlemen have severally favoured the public with, are so many striking examples of the efficacy of the practice. The OBJECTORS, in answer to this mass of evidence, appeal to the extensive trials of the stimulant practice made by Dr. Kentish and other respectable practitioners.

"Few men," (says the author of an Essay in your last Number) "have had more extensive opportunity of observation than Dr. Kentish; and it was only from a thorough conviction of its superiority," (the stimulant practice) "over the ordinary routine of practice, and this sanctioned by the superadded testimonies of many discriminating and unbiassed men, that he recommended this particular practice to the world. Since then, it has been progressively gaining ground, and is at this time followed in the metropolis by some professional men of the first rank and talents." By a similar train of observations, the most insignificant treatment of Burns and Scalds, amidst the almost infinite variety which prevails in modern practice, may be justified. For the bulk of our practitioners, so far from pursuing the stimulant treatment, make choice of remedies exceedingly different in their nature and effects. I allude to the common resources on these unfortunate occasions—Linimentum calcis—Aq. veg. min—Neutral ointment—and occasionally warm vinegar and chalk poultices. Thus we find the most feeble treatment sanctioned sanctioned by extensive practitioners, from what they term, long experience of its utility. But on a very little scrutiny it will be found, and indeed, candidly acknowledged, that they have been accustomed to follow up the same mode of treatment, even in all their cases. Whereas, if one remedy is not *absolutely good,* another may be *comparatively better;* and experience, however long and extensive, is generally inadequate to determine the point by any other means than *comparative trials;* and truly, long experience, on very many occasions, is but a cant phrase for no *experience to the purpose.*

Now, it is notorious that Dr. Kentish, according to his own documents at least, is totally unexperienced in the cold treatment, and yet he condemns it in opposition to all known experience, purely from hypothetical considerations. Assuredly, Dr. Kentish submitted the turpentine practice to comparative trials, with all the insufficient practice of the day! and the result of these experiments, I will allow, proved its pre-eminence over, what must be acknowledged, abominably bad practice; but by no means, which is the question at issue, could they, in the slightest manner, impeach the excellence of the cold treatment. It appears, that patients were suffered to linger exhausted from the severity of the accident. They were purged, under these circumstances, instead of being supported; and, when life was at its lowest ebb, a cordial draught was administered, and the patient expired!! (See Statement of the ordinary Treament in Dr. Kentish's First Essay.) That such was the most lamentable treatment, we are ready to allow; but it is both unfair and sophistical to consider its resemblance in any way with ours. The extent and severity of the local derangement is the only ascribable cause, cæteris paribus, of the alarming changes in the system which so frequently terminate fatally. We urge the imperious necessity of checking this local derangement, as expeditiously and effectually as possible. Dr. Kentish strenuously contends for the "*gradual restoration*" of the "healthy action;" and in consequence, affirms, that cold checks the local derangement TOO SOON, and in proof, alleges the fatal termination of cases, in which the local *derangement has not been checked at all!* Thus in your Journal, Gentlemen, Dr. Kentish in strong terms, pronounces the superior utility of his practice, and acknowledges "*that circumstances have very happily occurred to ascertain so important a point.*"

But why has the terebinthine treatment been preferred

 by by many other practitioners, and not the application of cold? The same observations which apply to Dr. Kentish's predilection for turpentine, apply also to practitioners in general who employ it. The same circumstances, which have imposed upon Dr. Kentish's judgement, have imposed also upon theirs. They think that warm turpentine is a MOST excellent remedy, and *infer*, contrary to experience, that cold water must be pernicious, as though the human mind had such influence over the agency of matter, that a *mere mental process* could possibly retort the *evidence of actual observation!* Yet such is the case before us, and the *dictates of indisputable facts* are opposed by the *force of imagination.*

We must not forget the influence of Dr. Kentish's theory; but independent of all bias from that source, in general it will be found, that the surgeons who hold turpentine in estimation, have on no occasion given the incessant application of cold any trial, or even consideration. They have been accustomed to treatment miserably deficient in the acute stage of these injuries, and to the same management, perhaps, which Dr. Kentish witnessed after the patient was exhausted by the severity of the symptoms. *Their gage of good treatment was obviously and highly fallacious,* and they were thus doubly operated upon by the sophistry of Dr. Kentish's inference, and the sophistry of their own. In confirmation of the preceding observations, I will only state the circumstance of the cold treatment, though adopted by a few individuals, being, till of late, altogether unnoticed in general practice; and further, those gentlemen, whom I have seen employ turpentine, and those whom I am acquainted with, that recommend it, all acknowledge their inexperience of the application of cold on similar occasions.

Before comparing the advantages and disadvantages of the stimulant and refrigerant practice, Icannot but observe, that Dr. Kentish's judgement must unavoidably be biassed by the theory he so firmly espouses. That practitioner must, indeed, be dull to daily occurrences, who cannot call to mind, numerous instances of perfectly nsignificant remedies being extolled as most powerful, and of decidely useful resources being condemned as unsafe; and all from the infatuating influence of theory. It is not therefore unaccountable, that Dr. Kentish's advantageous statement of his mode of treatment, appears overcharged, when compared with the observations of other surgeons, less interested in the result. From the opportunities tunities which I have had of witnessing the employment of turpentine, in one fatal, and in two or three recoverable cases, I considered the remedy as wholly insufficient in alleviating the torture in the acute stage; and in those cases which recovered, as far as the turpentine might affect the subsequent healing of the wound, I could not observe it superior to the common practice. Indeed, I fully concede to the observation, that the protraction of the healing process, is most frequently to be looked for in the constitution of the patient; and cases of daily occurrence go to prove, that the long continuance of the subsequent sore, could not possibly have been anticipated from the extent or situation of the wound, or nature of the remedies employed Several tedious cases of this kind have come under my inspection and management. I may further add, that the experience of some practitioners, which I have had the advantage of becoming acquainted with, teaches the insufficiency of the terebinthine treatment, in every particular for which it has been extolled. They made trial of the turpentine, but not finding its advantages to accord with their expectations, they continue to pursue their former method of cure.

Whilst attempting to appreciate the real importance of Dr. Kentish's practice, I may mention the material oversight betrayed in his deduction from the events of the three first cases, which he submitted to the comparative trials of different modes of treatment. Nothing is more indefinite than the extent of injury which shall occasion death, in different subjects; and in a like manner, nothing is more indefinite than the period at which the fatal event shall take place. But Dr. Kentish seems to think otherwise, and by overlooking idiosyncracy of constitution entirely, he unwarily considers the extent of the injury, and nature of the remedies, as circumstances which precisely regulate the progress and termination of the case; when, in fact, idiosyncracy of constitution is a very important circumstance concerned. The operations of nature being thus excluded, nothing can interfere with the conclusion, "that patients immersed in pure liquid fire, have by one mode of treatment been exhausted by pain and fever in the space of nine days; and that by another, existence was prolonged to the twelfth," (Observe the precision) "That by a third the patient was saved; and this, when *all other circumstances*, except the treatment, were the same." Tho' I think the first case affords an example of very bad treatment, yet I never can agree to the extent of these positions; for I apprehend it highly improbable, that the difference

 between between life and death, in the first and last cases, could warrantably be attributed to the additional resource in the one case of topical remedy, which must be acknowledged to posses little power in arresting the progress of the local derangement in the acute stage, to which the fatal termination must especially be traced. It is, in this manner, that undue confidence is so frequently placed in favourite remedies; their real or supposed operation intently occupies the mind, and they are referred to as the sine qua non of every good change which takes place. Thus IRON, a comparatively inert medicine, we see now meets with more encomiums than the great Boerhaave ever dared to bestow upon it. Boerhaave has always been considered, by sober practitioners, to have held too exalted notions of the virtues of this metal; but, it would appear, with great injustice; for carbonate of iron is, in these days, confidently recommended for the *cure of cancer!* *

To return. The insufficient effects of turpentine as a resource to abate the severity of the symptoms in the acute stage of burns and scalds, appears very strikingly, without examining its competition with the use of cold under similar circumstances. Dr. Kentish's cases themselves incontestably prove the long continuance of the torture after the injured parts have been dressed, and after having gradually decreased, of its frequent return in "paroxysms." In the third case it is minutely detailed, "that the pain had gradually abated in about *three hours, so as to be supportable.*" "Second day, pulse 120; has had several paroxysms of severe pain during the night; has also had some disturbed slumbers." "Third day, *easier* than before, pulse 120." I shall only further observe, that the reports of cases under the terebinthine treatment, in reference to the duration and return of the torture, would for the most part, mutatis mutandis, serve as reports for patients treated in the usual insufficient manner.

On the other hand, the decided advantage of cold applications in the acute stage of burns and scalds, is so immediate and remarkable, that cause and effect cannot be more

* I speak not against carbonate iron, as a palliative in cancerous affections, which do not admit of interference with the knife; but even under these circumstances, it must not diminish the importance of those means, by which we keep under the action of the vascular system: Abstemious regimen, which Douteau carried to such great extent; occasional laxatives, &c.; opium to diminish irritation, &c. I have seen the practice with iron adopted in cases of cancer of the uterus without any apparent advantage.

more pointedly connected. No tenet in medicine is perhaps better established than that the well-directed application of cold will constantly suppress, and frequently altogether remove, the local derangement immediate upon these injuries. If there is any meaning in the comparison of " a medicine acting like a charm," from what little I have had occasion to observe of the operation of cold on these occasions, I may consider it as an example. It is pleasing however to find, on a point of so much importance, that the correctness of the statement respecting the effects of cold, as to matter of fact, is in a great measure, I believe entirely, *acknowledged.* Dr. Kentish's irreconcilable hostility to the employment of cold is founded on the very fact, of the symptoms being so remarkably diminished by its application. He considers the symptoms (to wit, the extraordinary degree of action, the torture, the unnatural production of heat) not as the CAUSE, but as a PREVENTIVE of the extension of mischief. The increased action, he contends, must gradually subside into " healthy action." The spontaneous cravings of the sufferer for quietude must not be indulged. He calls patients the " dupes of their own feelings," because they very naturally consider the state of agony a very material part of the disease.

In consequence of the known extent of Dr. Kentish's practice on the injuries under consideration, his earnest recommendation of turpentine, exclusively, as a local remedy, I feel aware, has weighed much in the estimation of several surgeons, eminent for their professional talent and attainment. It is on this account I am the more anxious to be understood, that his Objections to the use of Cold are unfounded; that they are merely a part of a theory altogether erroneous; that they do not resolve themselves into ANY of his experience; and that they amount only to a mistaken notion.

It might have been expected that I should have entered formally into the merits of the Theory, particularly as in the Essay, dated Newcastle, in your last Number, it is observed, " As to the objections that have been adduced against the theory, most of them, I think, have originated in a defective acquaintance with it." I shall only reply, that the principles of it are contrary to all known facts.— If a sudden change of action, merely, could possibly produce " *torpor,*" a paroxysm of fever, or a slight fainting fit, must inevitably be mortal diseases. If the gradual abstraction of stimulus from burns or scalds was really re-

 quired quired to prevent "TORPOR," we should never have been called in to diminish "extraordinary action," long after the accident! The "extraordinary action" must have fallen into a state of "TORPOR" from the whole stimulus of "liquid fire" being entirely withdrawn. If it was necessary to replace the stimulus of scalding hot water, or, according to Dr. Kentish, of 200°, luke warm spirits of turpentine is by no means an equivalent stimulus; yet such practical errors Dr. Kentish adopts, from an inaccurate idea of the nature of the disease, frost-bitten; of the laws by which it is produced; and of its RELATION to ordinary stimuli. A moment's reflection ought to have shewn the futility of these confounded notions. Inflammation, we know, can continue and increase, independent of the original cause which produced it. This circumstance distinguishes inflammation from erythismus. So far from the fire which produced the disease being necessary, according to Dr. Kentish, to continue the action, or to prevent "TORPOR," the inflammation (and of course the action one of the symptoms) commences and increases AFTER the fire has been withdrawn, and FREQUENTLY A DISTINCT PAUSE OF EASE INTERVENES. Dr. Kentish must feel assured, that the mortification SUBSEQUENT to the first effects of the fire, is in consequence, and only in consequence, of the *continued* severity of the inflammation; and that whilst the symptoms of inflammation are most violent and severe, *mischief must extend,* although the injured parts *be smeared with turpentine;* and that a remedy, even if COLD, *which powerfully retards, and sometimes altogether removes, the symptoms of inflammation, must inevitably as powerfully retard or remove the tendency to destruction.*

I have confined myself entirely to the consideration of the acute stage of burns and scalds, because, as every one must feel convinced, in the general run of cases, it is the most important period of the accident, whether we regard the sufferings of the patient, the consequent mortification of parts, or the future danger from symptomatic irritation. The object to command the inflammation concerns the whole period of the disease, for according to its severity and duration, cæteris paribus, the extent of the local and general derangement depends. After this endeavour to clear up the truth, I may be allowed to conclude, that,—The incessant application of cold, in the acute stage of burns and scalds, according to all known experience yet made made public, is the only remedy employed that can powerfully fulfil the important indication of diminishing and removing the inflammation immediate upon these accidents; and that all objections yet advanced, resolve themselves into crude conjectures.

I am, &c.

